# Factors predicting the adhesion and prolonged lost days of work in patients with extensor tendon adhesion of the hand

**DOI:** 10.3389/fsurg.2024.1304202

**Published:** 2024-05-01

**Authors:** Gong Xiao, Jinzhong Wang, Ningjie Zhang, Juanjuan Hao

**Affiliations:** ^1^ Shaoxing Hospital of Traditional Chinese Medicine, Shaoxing, Zhejiang, China; ^2^ School of Medicine, Shaoxing University, Shaoxing, China

**Keywords:** extensor tendon repair, hand, tendon adhesion, prolonged lost days of work, triglyceride level

## Abstract

**Objective:**

Extensor tendon adhesion receive less attention recently. This study aims to analyze influencing factors of adhesion and prolonged lost days of work in patients with extensor tendon adhesion of the hand.

**Method:**

We performed a retrospective study in patients with extensor tendon injuries who underwent primary surgical repair and early rehabilitation. We observed the differences between non-tendon adhesion and adhesion patients after surgical repair, and used the receiver operating characteristic curve to distinguish them. Then we explored the influencing factors of adhesion. In addition, we studied the lost days of work and the influencing factors.

**Results:**

A total of 305 patients were included. 24.6% patients appeared tendon adhesion and the mean lost days of work was 12 weeks. MHISS scores, VAS scores, occupation and blood triglyceride level were the influencing factors of adhesion. The adhesion patients have increased MHISS scores (*p* < 0.001), VAS scores (*p* < 0.001), blood triglyceride levels (*p* < 0.001) and lost days of work (*p* < 0.001) than non-tendon adhesion. The optimal cut-off value of blood triglyceride level to distinguish non-tendon adhesion from adhesion was 1.625 mml/L, and MHISS scores was 20.5. Smoking, MHISS scores, blood triglyceride levels were the influencing factors of lost days of work in adhesion patients. There was positive correlation between lost days of work and triglyceride level (*r* = 0.307, *p* = 0.007), and MHISS scores (*r* = 0.276, *p* = 0.016).

**Conclusion:**

To minimize the occurrence of adhesion, doctors should pay attention to patients with higher MHISS and VAS scores, blood triglyceride levels, especial for the blue-collar and unemployed one. High triglyceride level may be a new influencing factor.

## 1 Introduction

The tendon injury represents the second most common injury of the hand (after fractures) (1). The extensor tendon injury accounts for 20% of the whole flexor and extensor tendon injury of the hand. Extensor tendons are susceptible to damage because of their relatively thin and soft tissue coverage (1). Early treatment of extensor tendon injury is essential to clinical outcome (2–9). If tendon laceration or anomalies exist in the extensor mechanism, surgical repair is warranted (10, 11). The formation of tendon postoperative adhesion is a common clinical complication (12). Postoperative adhesion is a natural process of surgical wound healing. However, unsuitable postoperative adhesion can cause adverse effects. Tendon adhesion can restrict tendon movements such as tendon gliding and joint flexion.

As the sliding range of the extensor tendon was smaller than that of the flexor tendon, and most of the tendons were located in the subcutaneous tissue, surgical doctors usually think that extensor tendon postoperative adhesion has less effect on mobility and movement of hands. However, current clinical practice found that the incidence of extensor tendon adhesion is increasing, and treatments often fall into the vicious cycle of adhesion–release–adhesion, which seriously affects the functional rehabilitation of patients. As patients are usually working people, adhesion prolongs the lost days of work and produces the substantial burden to the individual and society.

Extensor mechanism of hand is extremely intricate (13). But extensor tendon injuries and adhesion receive less attention than flexor tendon in the literature recently. A better understanding of the variables that may contribute to the adhesion is helpful in predicting the risks of poor outcomes and determining their proper management. Several individual, injury, and work-related factors may be associated with the adhesion (14).

Dyslipi demia (DLD) encompasses disorders of lipoprotein metabolism, including overproduction of TC (i.e., hyperlipidemia), low-density lipoprotein (LDL), and triglycerides (TGs) and/or underproduction of HDL. Current evidence suggests that lipids can accumulate in the extracellular matrix of tendons, leading to the formation of lipid deposits altering mechanical properties and increasing local inflammation (15, 16). Animal models display reduced tendon elasticity, greater fatty infiltration, and poor tendon-to bone healing (17–19). DLD is shown to be an independent risk factor for tendon pathology. Recent studies reported that metabolic factors play a role in the tendon injury (20). For example, Louis J. Soslowsky reported that hypercholesterolemia has a detrimental biomechanical effect on tendon healing in our rat rotator cuff injury and repair model (21). It implies that we should use a more comprehensive perspective and pay much attention to the metabolic status of patients during tendon injury prevention. Actually, it has never been explored in the tendon adhesion and even extensor tendon adhesion till now. Our study would firstly take the metabolic factors (such as triglycerides and cholesterol levels) into account.

This study firstly identified the sociodemographic and injury characteristics of patients with extensor tendon injury of the hand and has undergone the tendon repair with a four-strand modified Kessler core suture. We observed the different features between the non-tendon adhesion and tendon adhesion patients after surgical repair, and used the receiver operating characteristic (ROC) curve to distinguish them. Then we explored the influencing factors for the occurrence of adhesion. With main interest in the metabolic factors, other objective indicators (such as blood calcium levels), individual, injury, and work-related factors would be considered together. In addition, our study studied the lost days of work and the influencing factors. Here we present our findings.

## 2 Materials and methods

A retrospective study was conducted, which included patients with the extensor tendon injuries from January 2013 to June 2021. All patients who underwent primary extensor tendon injury repair using a four-strand modified Kessler core suture and early rehabilitation by an experienced surgical team, and who had at least a 12-month follow-up, were identified from medical records. Exclusion criteria were severe comorbidities that may have extensive influences on the lost days of work, phalangeal fractures, nerve injuries, amputations, replantation, non-adherence to treatment and incomplete data. Sociodemographic characteristics included age at injury, gender, smoking/alcoholic status and occupation status (blue-collar, white-collar and non-employment groups). The clinical and injury characteristics included the cause of injury, zone of injury, side of the injured hand, number of involved tendons, distribution of fingers and tendons affected, severity of injury, time of surgery, and number of lost days of work. The objective laboratory indicators included white blood cell level (WBC), albumin level, triglyceride level, cholesterol level, calcium level, etc. All related clinical information was collected.

The severity of the injury: the Modified Hand Injury Severity Score (MHISS) was used to evaluate the severity of the injury (22). Patient medical notes of the injury that were recorded upon arrival at the emergency room, and those of the findings during surgery, were reviewed. The MHISS value was classified as minor (<20), moderate (21–50), severe (51–100), or major (>101).

### 2.1 Surgical technique

**Figure 1:**
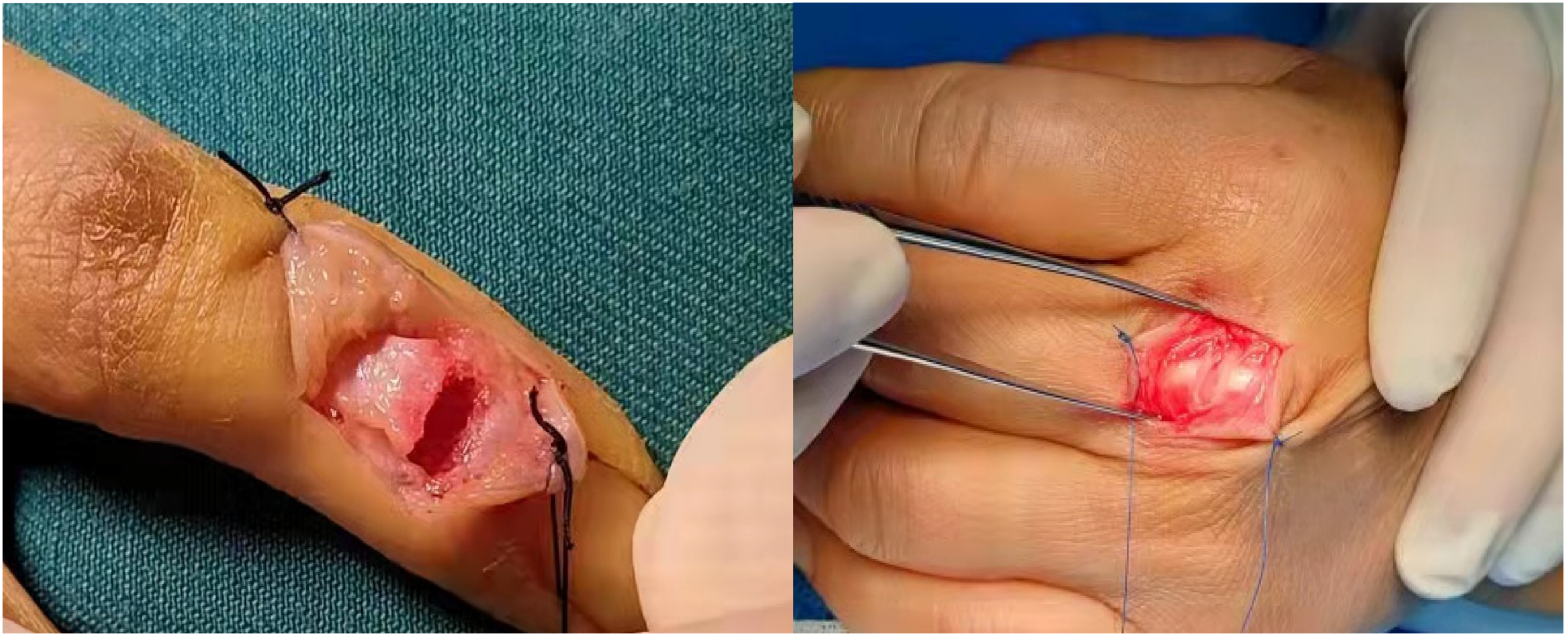
Typical extensor tendon injury.

All extensor tendon repairs were carried out under general anesthesia, local anesthesia, or brachial plexus anesthesia using a four-strand modified Kessler technique with 4-0 polypropylene (Figure 1). Rehabilitation Protocol: After surgery, use a volar splint, immobilize the wrist in 30°–40° extension while using a rubber band to stretch all interphalangeal joints. In the splint, the fingers should be actively flexed, and passively stretched by elastic traction. Passive flexion and active finger extension are prohibited. In the first three weeks after surgery, the patients were instructed to perform active flexion and passive extension of the interphalangeal joints. After the stitches are removed, massage the incision area. Active finger hook flexion and fist formation in the splint started at the end of week 3; passive finger extension exercises by elastic. The splint was removed at the end of week 6 and active finger extension was started. After week 7, start resistance exercise. Continue with edema control and scar massage as needed.

### 2.2 Statistics

The SPSS 25.0 program (IBM Corporation, Armonk, NY, USA) was used for the analysis. Sociodemographic and injury characteristics were reported using descriptive statistics (mean, standard deviation, range, number, and percentage). The Mann–Whitney *U* test, Student's *t*-test, and Chi-squared were used to compare continuous or nominal variables between groups. A binary logistic regression model was used to estimate influencing factors on tendon adhesion. The optimal cut-off value was to distinguish tendon adhesion from non-tendon adhesion using a receiver operating characteristic (ROC). The maximum Youden index method was used to determine the best critical value. A multiple linear regression model was used to estimate influencing factors on the lost days of work. Spearman correlation was used for relationship between clinical features. Statistical significance was determined when *p*-value < 0.05.

## 3 Results

### 3.1 Patient characteristics and injury characteristics

A total of 305 extensor tendon injury patients were included in this study. Patients characteristics were shown in Table 1. Overall, the mean age was 46.53 ± 14.45 years, 237 (77.7%) patients were male, 159(52.1%) patients had Smoking history, and 22(7.2%) patients were alcoholics. Blue-collar workers (65.9%) occupied the largest number of patients.

**Table 1:** Patient characteristics and injury characteristics.

	Overall patients	Non-tendon adhesion patients	Tendon adhesion patients
*n*	305	230	75
Gender: Female/Male	68/237	54/176	14/61
Age (Mea*n* ± SD), y	46.53 ± 14.45	46.86 ± 14.63	45.53 ± 13.94
Smoking history, *n* (%)	159 (52.1%)	122 (53.0%)	37 (49.3%)
Alcoholic, *n* (%)	22 (7.2%)	14 (6.1%)	8 (10.7%)
Occupation			
White-collar workers, *n* (%)	20 (6.6%)	17 (7.4%)	3 (4.0%)
Blue-collar workers, *n* (%)	201 (65.9%)	169 (73.5%)	32 (42.7%)
Unemployed, *n* (%)	84 (25.5%)	44 (19.1%)	40 (53.3%)
Cause of injury			
Crush injury *n* (%)	50 (16.4%)	35 (15.2%)	15 (20.0%)
Cut wound, *n* (%)	169 (55.4%)	129 (56.1%)	40 (53.3%)
Zone 1 injury, *n* (%)	100 (32.8%)	71 (30.9%)	29 (38.7%)
Zone 2 injury, *n* (%)	93 (30.5%)	76 (33.0%)	17 (22.7%)
Zone 3 injury, *n* (%)	91 (29.8%)	65 (28.3%)	26 (34.7%)
Number of involved tendons (Mean ± SD)	1.12 ± 0.5	1.12 ± 0.5	1.11 ± 0.5
The affected thumb, *n* (%)	280 (91.8%)	210 (91.3%)	70 (93.3%)
Time to surgery after injury (Median, IQR), d	1 (1–3)	1 (1–4)	1 (1–1)
Time of surgery (Median, IQR), min	35 (30–45)	35 (30–450)	35 (30–50)
Lost days of work (Median, IQR), w	7 (4–12)	6 (4–8)	12 (7–18)
MHISS scores (Median, IQR)	26 (18–31.5)	26 (15–30)	30 (25–36)
VAS scores (Mean ± SD)	2.8 ± 0.6	2.71 ± 0.66	3.08 ± 0.27
Wound type			
Open wound, *n* (%)	200 (65.6%)	145 (63.0%)	55 (73.3%)
Closed wound, *n* (%)	10,534.4(%)	85 (37.0%)	20 (26.7%)
White blood cell levels (Median, IQR), ng/ml	6.4 ± 2.45	6.73 ± 2.38	6.95 ± 2.63
Blood calcium level (Mean ± SD), mmol/L	2.28 ± 0.12	2.29 ± 0.12	2.27 ± 0.1
Blood albumin level (Mean ± SD), g/L	45.3 ± 3.16	45.3 ± 3.10	45.6 ± 3.35
Blood triglyceride level (Median, IQR), mmol/L	1.26 (0.84–2.02)	1.71 (1.03–3.42)	1.2 (0.81–1.69)
Blood cholesterol level (Mean ± SD), mmol/L	4.69 ± 0.98	4.63 ± 0.99	4.88 ± 0.93

MHISS, modified hand injury scoring system; VAS, visual analogue scale; IQR, interquartile range; SD, standard deviation; y, year; w, week; d, day; min, minute.

The most common cause of tendon injury is cut wound (55.4%). Zone 1 injuries were most common (32.8%), followed by zone 2 (30.5%). The thumb was most frequently affected (91.8%), The mean number of involved tendons was 1.12. The mean MHISS scores was 26(range 18–31.5). A total of 75 patients (24.6%) appeared tendon adhesions after the primary repair. The mean lost days of work (delayed working time due to injury) was 7 weeks (range 4–12).

### 3.2 Influencing factors of tendon adhesion by the logistic regression model

To predict influencing factors of tendon adhesion, we used the logistic regression model. In this model, the occurrence of tendon adhesion was the dependent variable. A range of predictor variables covers three main domains: Firstly, non-tendon injury related factors were included. They were gender, age, etc. Secondly, factors reflecting injury severity were included: MHISS scores, VAS scores, number of involved tendons, etc. Lastly, we included the objective indicators (such as blood triglyceride level). The result of the logistic regression model was given in Table 2, MHISS scores, VAS scores, occupation and blood triglyceride level were the influencing factors.

**Table 2:** Summary of the logistic regression model of tendon adhesions.

Variable	Unstandardized regression coefficients (95% CI)	Standard error	*p* value
Gender			
Male (reference)	(Reference)	(Reference)	
Female	0.737 (0.743–5.879)	0.528	0.162
Age	−0.026 (0.943–1.008)	0.017	0.131
Occupation			
Unemployed (reference)	(Reference)	(Reference)	
White-collar workers	−2.278 (0.020–0.536)	0.844	0.007
Blue-collar workers	−2.025 (0.063–0.276)	0.377	<0.001
Smoking history			
Smoking history (reference)	(Reference)	(Reference)	0.447
Non-smoking history	0.194 (0.395–3.739)	1.371	
Alcoholic			
Alcoholic (reference)	(Reference)		
Non-alcoholic	−1.150 (0.089–1.131)	0.649	0.077
Time to surgery after injury (day)	0.003 (0.957–1.052)	0.024	0.894
Time of surgery (min)	0.015 (0.994–1.036)	0.011	0.175
Number of involved tendons	−0.205 (0.360–1.848)	0.418	0.624
MHISS scores	0.060 (1.025–1.100)	0.018	0.001
VAS scores	2.274 (3.621–26.09)	0.504	<0.001
Blood white blood cell level (ng/ml)	−0.044 (0.824–1.113)	0.077	0.569
Blood albumin level (g/L)	−0.028 (0.848–1.114)	0.070	0.687
Blood triglyceride level (mmol/L)	0.418 (1.144–2.016)	0.145	0.004
Blood Calcium level (mmol/L)	−2.511 (0.002–4.263)	2.021	0.214
Blood Cholesterol level (mmol/L)	0.144 (0.784–1.702)	0.198	0.465

MHISS, modified hand injury scoring system; VAS, visual analogue scale.

### 3.3 Comparison between non-tendon adhesion and tendon adhesion patients

**Figure 2:**
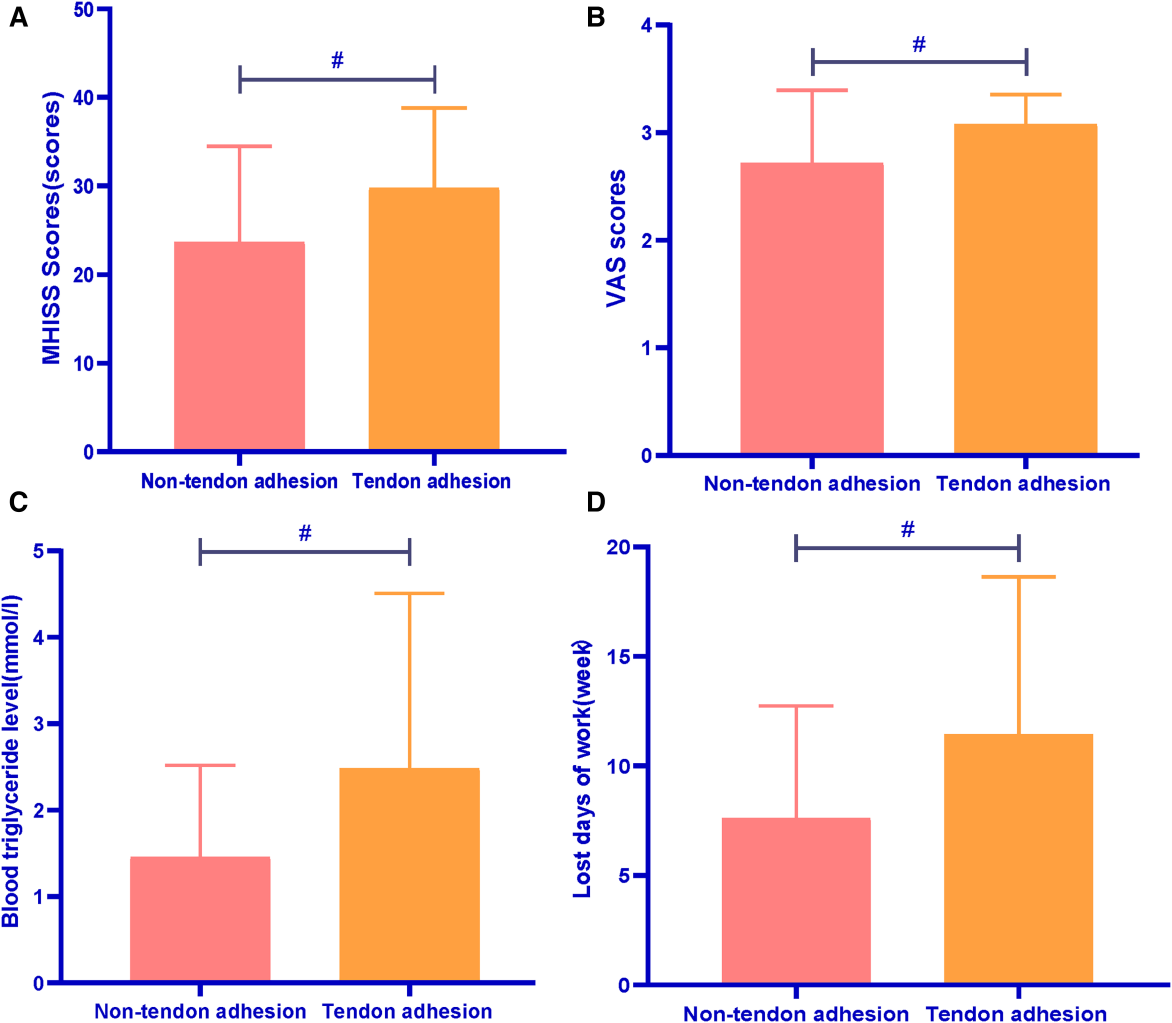
Comparison between Non-tendon adhesion and tendon adhesion patients. #*p* < 0.01. (**A**) Comparison of the MHISS scores between the two groups; (**B**) comparison of the VAS scores between the two groups; (**C**) comparison of the Blood Triglyceride levels between the two groups; (**D**) comparison of the lost days of work between the two groups.

Compared with the non-tendon adhesion patients, MHISS scores, VAS scores, Blood Triglyceride levels and lost days of work were increased in the tendon adhesion patients, and the difference was statistically significant (*p* < 0.001, *p* < 0.001, *p* < 0.001, *p* < 0.001 respectively). It was shown in Figure 2.

### 3.4 ROC curve for non-tendon adhesion and tendon adhesion

The optimal cut-off value was to distinguish Tendon adhesion using a receiver operating characteristic (ROC). The optimal cut-off value of Blood Triglyceride level to distinguish non-tendon adhesion from tendon adhesion was 1.625 mml/L, with sensitivity of 86.7% and specificity of 42.2%. The optimal cut-off value of MHISS scores to distinguish non-tendon adhesion from Tendon adhesion was 20.5, with sensitivity of 58.7% and specificity of 73.5%.

**Figure 3:**
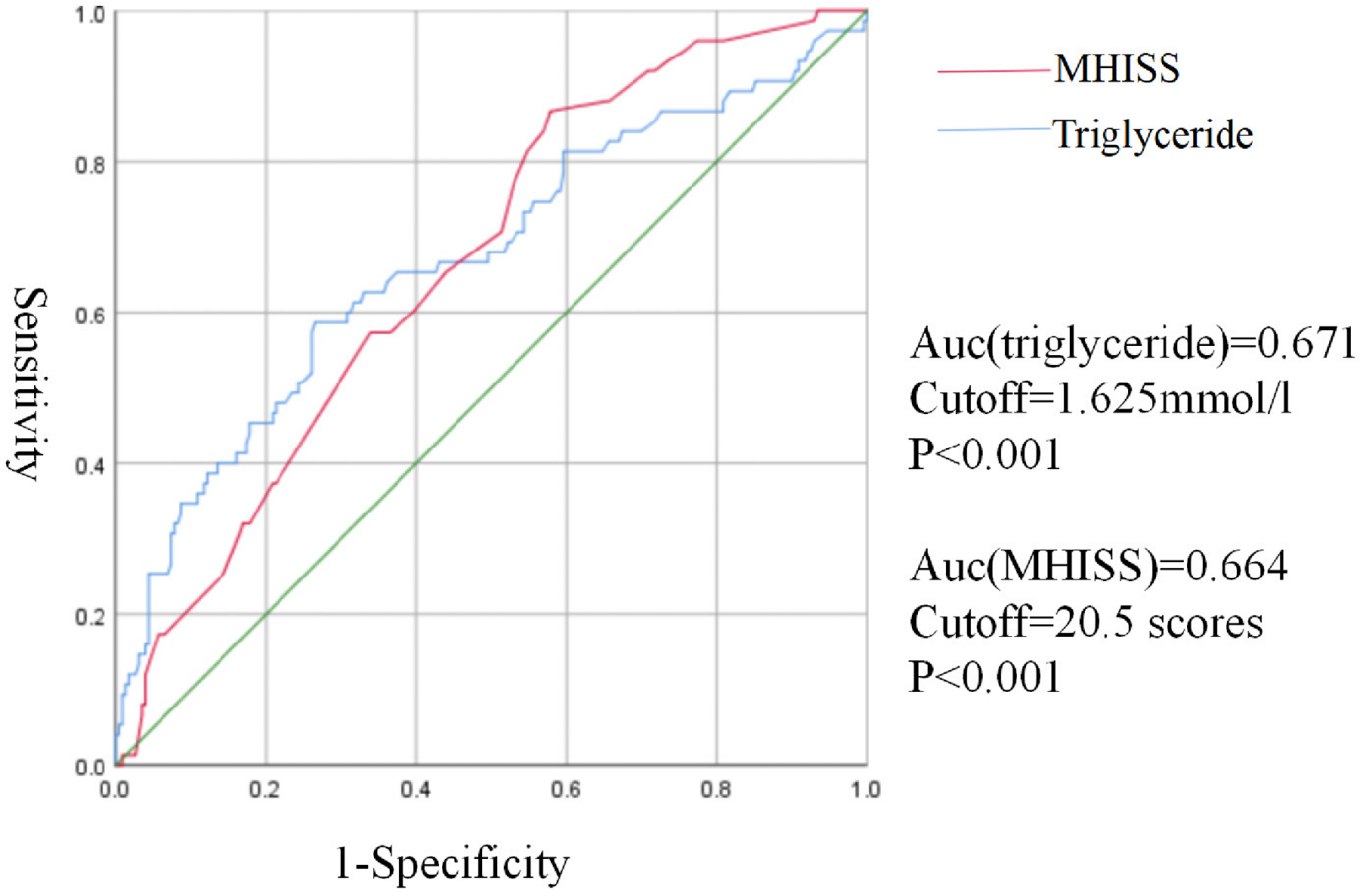
ROC curve for non-tendon adhesion and tendon adhesion.

The result was shown in Figure 3.

### 3.5 Influencing factors of lost days of work in tendon adhesion patients

To predict influencing factors of Lost days of work in Tendon adhesion patients, we used a multiple regression model. In this model, Lost days of work was the dependent variable. A range of predictor variables: gender, age, MHISS scores, VAS scores, number of involved tendons, White Blood Cell level, Blood Calcium level, Albumin level, Blood Triglyceride level and Blood Cholesterol level, etc. The result of the multiple linear regression model was given in Table 3. Smoking, MHISS scores, Blood Triglyceride level were the influencing factors. Other factors did not affect Lost days of work in Tendon adhesion patients observed in this study.

**Table 3:** Summary of the multiple linear regression model of lost days of work in tendon adhesion patients.

Variable	Unstandardized regression coefficients (95% CI)	Standard error	*p* value
Current smoker			
Smoker (reference)	(Reference)	(Reference)	
Non-smoker	−5.524 (−10.448 to −0.600)	2.462	0.029
Blood triglyceride level (mmol/L)	1.015 (0.036 to 1.994)	0.489	0.042
MHISS scores	0.275 (0.074 to 0.476)	0.100	0.008

MHISS, modified hand injury scoring system; CI, confidence interval.

**Figure 4:**
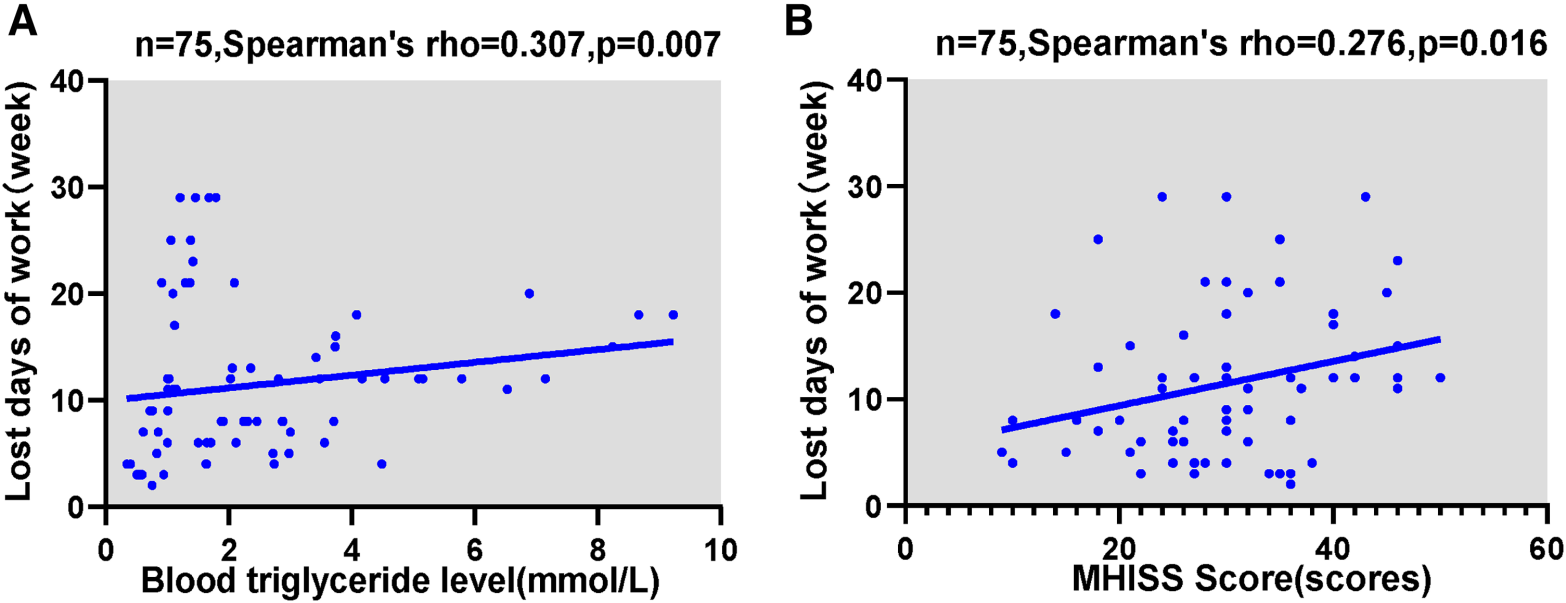
Correlation analyses in tendon adhesion patients. (**A**) Correlation between the lost days of work and Triglyceride level; (**B**) correlation between the lost days of work and MHISS scores.

Further correlation analysis showed that there was significant positive correlation between lost days of work and Triglyceride level (*r* = 0.307, *p* = 0.007), and MHISS scores (*r* = 0.276, *p* = 0.016). It was shown in Figure 4.

### 3.6 Another correlation analyses among the clinical features in the entire group of tendon injury patients

**Figure 5:**
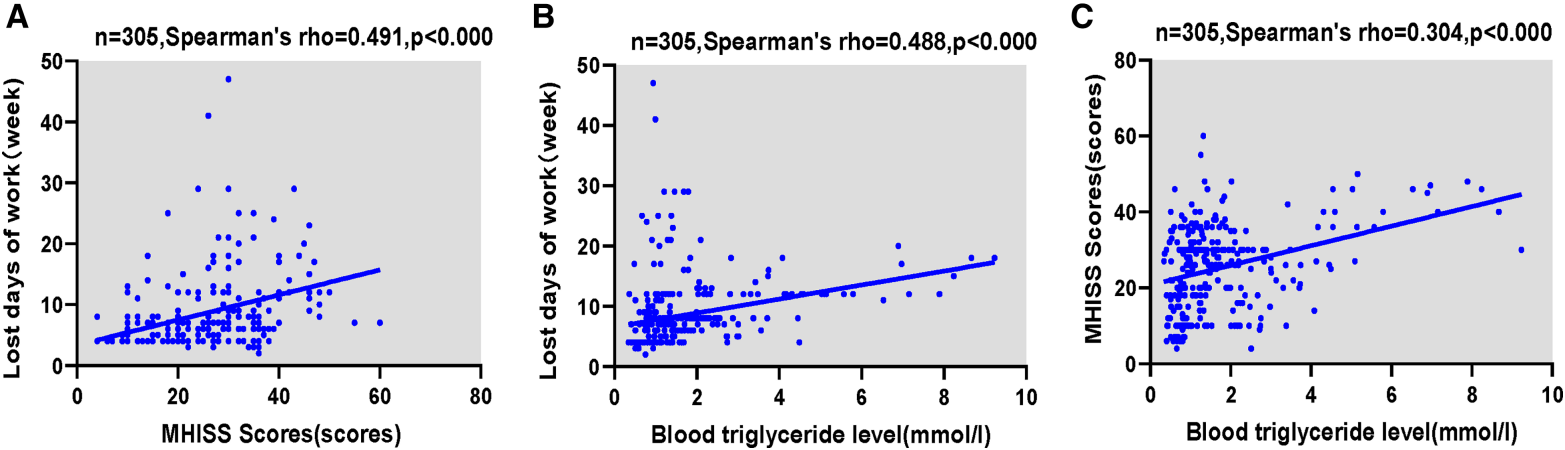
Correlation analyses in the whole tendon injury patients. (**A**) Correlation between the lost days of work and MHISS scores; (**B**) correlation between the lost days of work and Triglyceride level; (**C**) correlation between the MHISS scores and Triglyceride level.

We still performed a correlation analysis in the entire group of tendon injury patients. It showed that there was significant positive correlation between lost days of work and Triglyceride level (*r* = 0.488, *p* *< *0.000), and MHISS scores(*r* = 0.491, *p* *< *0.000). And we found there was significant positive correlation between MHISS scores and Triglyceride level (*r* = 0.304, *p* *< *0.000). It was shown in Figure 5.

## 4 Discussion

We conducted the present study to provide a better understanding of the extensor tendon adhesion of hand, and the potential contributors to adhesion. This study was observed by a retrospective study for the first time. In the analysis, we estimated the rate of adhesion formation to be 24.6%. We found MHISS scores, VAS scores, occupation and blood triglyceride levels were the influencing factors of tendon adhesion. The tendon adhesion patients have higher MHISS scores, VAS scores and blood triglyceride levels than non-tendon adhesion patients. Especially the blood triglyceride level and MHISS scores can provide reference value to distinguish non-tendon adhesion from tendon adhesion.

The extensor tendons are flat, thin, and broad, making them more prone to injury. The extensor tendons on the back of the hand are superficial, have a high injury rate, and are prone to adhesion to bone. Compared to the flexor tendons, the extensor tendons are weaker and tend to be overstretched during active movement. Adhesion may occur when the tendons are injured for any reason. In patients with severe tendon injuries, it is difficult to avoid tendon adhesion after surgical repair. It may affect the sliding of the tendon, even lead to the failure of the repair surgery. Patients with higher VAS scores may be related to the severity of the tendon injury. On the other hand, patients would be afraid of early functional exercises because of the pain, which provides conditions for the formation of adhesion. Due to the nature of work, blue-collar workers and unemployed people are more prone to tendon injury than white-collar workers, as well as tendon adhesion after surgical repair. When the triglyceride level was higher, the blood flow would be slow, and the blood circulation of the tendon would be affected, which affects the heal of the tendons. Then the body needs to establish collateral circulation from surrounding tissues to obtain nutrients, which may be another cause of adhesion. In recent years evidence has emerged for metabolic risk profiles to play a role in the development of tendon injury. This increased risk of tendinopathy and tendon ruptures may be due to overload caused by obesity. Excessive fat promotes a release of cytokines that might influence tendon metabolism or response to microtrauma. Due to the limited sample size, we have not found the abnormalities in cholesterol levels. But the abnormal triglyceride levels also provide evidence that metabolism may affect tendon adhesion.

In our study, the tendon adhesion patients also have longer lost days of work, and we found smoking, MHISS scores, blood triglyceride level were the influencing factors. Further analysis showed that there was positive correlation between lost days of work and triglyceride level and MHISS scores. We still performed a correlation analysis in the entire group of tendon injury patients. It showed that there was significant positive correlation between lost days of work and triglyceride level and MHISS scores. Previous studies have suggested that the recovery time for flexor tendon injuries is 50 days (23). In our study, the mean time to return to work was 7 weeks for patients with extensor tendon injuries, 6 weeks for non-adhesion patients, 12 weeks for patients with tendon adhesions. In this study, we analyzed the reasons for the prolonged lost days of work in patients with tendon adhesions. Besides MHISS scores and Blood Triglyceride level, smoking was also one of the risk factors. Smoking increases the concentration of carbon monoxide in the blood, resulting in ischemic damage to vascular endothelial cells. Smoking can make plasma low-density lipoprotein easily oxidized, which can promote the migration of blood monocytes into the endothelium and transform into foam cells. Foam cells cause hardening of the arteries, affect the tendon metabolism and blood circulation, and the tendon healing.

However, our findings are based on a relatively small number of only 305 patients. The sample size was too small to confirm the validity of our conclusions. Secondly, the data was obtained from a single center; therefore, it is difficult to generalize our results. In the future, we will verify the conclusion in a large-sample, multi-center clinical trial and explore the mechanism of tendon adhesion in animal model experiments.

To minimize the occurrence of adhesion, surgical doctors should pay more attention to patients with high MHISS and VAS scores, blood triglyceride levels, especial for the blue-collar workers and unemployed one. High triglyceride level may be a new influencing factor.

## Data Availability

The raw data supporting the conclusions of this article will be made available by the authors, without undue reservation.

## References

[1] Yoon AP Chung KC. Management of acute extensor tendon injuries. Clin Plast Surg. (2019) 46(3):383–91. 10.1016/j.cps.2019.03.004 31103083

[2] Snow JW. A method for reconstruction of the central slip of the extensor tendon of a finger. Plast Reconstr Surg. (1976) 57(4):455–9. 10.1097/00006534-197604000-00007 1273126

[3] Maitra A Dorani B. The conservative treatment of mallet finger with a simple splint: a case report. Arch Emerg Med. (1993) 10(3):244–8. 10.1136/emj.10.3.244 8216604 PMC1285998

[4] Tetik C Gudemez E. Modification of the extension block Kirschner wire technique for mallet fractures. Clin Orthop Relat Res. (2002) 404:284–90. 10.1097/00003086-200211000-00043 12439271

[5] Handoll HH Vaghela MV. Interventions for treating mallet finger injuries. Cochrane Database Syst Rev. (2004) 3:CD004574. 10.1002/14651858.CD004574.pub2 PMC13255240 15266538

[6] Woo SH Tsai TM Kleinert HE Chew WY Voor MJ. A biomechanical comparison of four extensor tendon repair techniques in zone IV. Plast Reconstr Surg. (2005) 115(6):1674–81. discussion 1682–3. 10.1097/01.PRS.0000161463.83102.85 15861073

[7] Teoh LC Lee JY. Mallet fractures: a novel approach to internal fixation using a hook plate. J Hand Surg Eur Vol. (2007) 32(1):24–30. 10.1016/j.jhsb.2006.09.007 17134796

[8] Zubović A Egan C Sullivan M. Augmented (Massachusetts general hospital) Becker technique combined with static splinting in extensor tendons repairs zones III to VI: functional outcome at three months. Tech Hand Up Extrem Surg. (2008) 12(1):7–11. 10.1097/BTH.0b013e318123769e 18388749

[9] Lee SK Dubey A Kim BH Zingman A Landa J Paksima N. A biomechanical study of extensor tendon repair methods: introduction to the running-interlocking horizontal mattress extensor tendon repair technique. J Hand Surg Am. (2010) 35(1):19–23. 10.1016/j.jhsa.2009.09.011 20117304

[10] Chow JA Dovelle S Thomes LJ Ho PK Saldana J. A comparison of results of extensor tendon repair followed by early controlled mobilisation versus static immobilisation. J Hand Surg Br. (1989) 14(1):18–20. 10.1016/0266-7681(89)90005-3 2926213

[11] Chester DL Beale S Beveridge L Nancarrow JD Titley OG. A prospective, controlled, randomized trial comparing early active extension with passive extension using a dynamic splint in the rehabilitation of repaired extensor tendons. J Hand Surg Br. (2002) 27(3):283–8. 10.1054/jhsb.2001.0745 12074620

[12] Dy CJ Hernandez-Soria A Ma Y Roberts TR Daluiski A. Complications after flexor tendon repair: a systematic review and meta-analysis. J Hand Surg Am. (2012) 37(3):543–551.e1. 10.1016/j.jhsa.2011.11.006 22317947

[13] Matzon JL Bozentka DJ. Extensor tendon injuries. J Hand Surg Am. (2010) 35(5):854–61. 10.1016/j.jhsa.2010.03.002 20439000

[14] Çalışkan Uçkun A Yurdakul FG Ergani HM Güler A Yaşar B Başkan B Factors predicting reoperation after hand flexor tendon repair. Ulus Travma Acil Cerrahi Derg. (2020) 26(1):115–22. 10.14744/tjtes.2019.92590 31942748

[15] Hast MW Abboud JA Soslowsky LJ. Exploring the role of hypercholesterolemia in tendon health and repair. Muscles Ligaments Tendons J. (2014) 4(3):275–9. 10.32098/mltj.03.2014.02 25489542 PMC4241415

[16] Yang Y Lu H Qu J. Tendon pathology in hypercholesterolaemia patients: epidemiology, pathogenesis and management. J Orthop Translat. (2019) 16:14–22. 10.1016/j.jot.2018.07.003 30723677 PMC6350019

[17] Beason DP Abboud JA Kuntz AF Bassora R Soslowsky LJ. Cumulative effects of hypercholesterolemia on tendon biomechanics in a mouse model. J Orthop Res. (2011) 29(3):380–3. 10.1002/jor.21255 20939036

[18] Chung SW Kim JY Kim MH Kim SH Oh JH. Arthroscopic repair of massive rotator cuff tears: outcome and analysis of factors associated with healing failure or poor postoperative function. Am J Sports Med. (2013) 41(7):1674–83. 10.1177/0363546513485719 23631883

[19] Barth J Andrieu K Fotiadis E Hannink G Barthelemy R Saffarini M. Critical period and risk factors for retear following arthroscopic repair of the rotator cuff. Knee Surg Sports Traumatol Arthrosc. (2017) 25(7):2196–204. 10.1007/s00167-016-4276-x 27522591

[20] Skovgaard D Siersma VD Klausen SB Visnes H Haukenes I Bang CW Chronic hyperglycemia, hypercholesterolemia, and metabolic syndrome are associated with risk of tendon injury. Scand J Med Sci Sports. (2021) 31(9):1822–31. 10.1111/sms.13984 33963621

[21] Beason DP Tucker JJ Lee CS Edelstein L Abboud JA Soslowsky LJ. Rat rotator cuff tendon-to-bone healing properties are adversely affected by hypercholesterolemia. J Shoulder Elbow Surg. (2014) 23(6):867–72. 10.1016/j.jse.2013.08.018 24295837 PMC4029875

[22] Özgen M Merve Aydoğan A Uygur A Armağan O Berkan F Mutlu F. Rehabilitation cost share and cost analysis of traumatic hand injuries: our single-center results. Turk J Phys Med Rehabil. (2021) 67(3):308–14. 10.5606/tftrd.2021.5457 34870117 PMC8606991

[23] Nichols A Best KT Loiselle AE. The cellular basis of fibrotic tendon healing: challenges and opportunities. Transl Res. (2019) 209:156–68. 10.1016/j.trsl.2019.02.002 30776336 PMC6545261

